# Common features of Budd Chiari syndrome in Sudanese population: a computed tomography-based review and descriptive analysis

**DOI:** 10.3389/fmed.2025.1552366

**Published:** 2025-10-27

**Authors:** Abdoelrahman Hassan Ali Bakry, Auis Bashir, Nora Almuqbil, Asma Alamin, Ibtisam Abdallah Fadulelmulla, Abdullah G. M. ALMansour, Awatif M. Omer, Hozaifa Hassan Bairam, Abdelmoneim Sulieman, Zuhal Y. Hamd

**Affiliations:** ^1^Department of Radiotherapy, College of Medical Radiologic Sciences, Sudan University of Science and Technology, Khartoum, Sudan; ^2^Faculty of Medicine, Al Neelain University, Khartoum, Sudan; ^3^Department of Radiologic Technology, College of Applied Medical Science, Qassim University, Buraydah, Saudi Arabia; ^4^Department of Radiological Sciences, College of Health and Rehabilitation Sciences, Princess Nourah bint Abdulrahman University, Riyadh, Saudi Arabia; ^5^Department of Diagnostic Radiology, College of Applied Medical Sciences, University of Ha’il, Ha’il, Saudi Arabia; ^6^Radiology and Medical Imaging Department, College of Applied Medical Sciences, Prince Sattam Bin Abdulaziz University, Alkharj, Saudi Arabia; ^7^Department of Diagnostic Radiology, College of Applied Medical Sciences, Taibah University, Al-Madinah Al-Munawwarah, Saudi Arabia; ^8^Department of Diagnostic Radiology and Medical Imaging, Mubarak Al-Kabir Hospital, Kuwait, Kuwait; ^9^Department of Radiological Sciences, College of Applied Medical Sciences, King Saud Bin Abdulaziz University for Health Sciences, Riyadh, Saudi Arabia; ^10^Imaging Physics Section, Biomedical Physics Department, King Faisal Specialist Hospital and Research Centre, Riyadh, Saudi Arabia

**Keywords:** Budd-Chiari syndrome, computed tomography, hepatomegaly, ascites, liver, abdomen, hepatic veins

## Abstract

**Purpose:**

Different hospitals in Sudan detected a rare condition of liver vascular abnormalities characterized by vascular outflow impairment. The study aimed to describe the common radiological features of Budd Chiari syndrome and determine which feature is most frequently employed to characterize this condition during imaging techniques, primarily contrast-enhanced computed tomography (CECT) scans.

**Materials and methods:**

The study was conducted between March 2023 and June 2024 at Kuwaiti Specialized Hospital (KSH) and other diagnostic centers using a liver protocol on a CT machine (Optima 520 GE-CT machine, 16 slices). The study was a retrospective, cross-sectional, and review-based analysis of a rare study type carried out to characterize the state of BCS in Sudan; the CT scan’s findings on the liver, HVs, IVC, and abdomen were carefully assessed. The age range of the 61 patients who underwent a successful triphasic CT abdomen for the liver was 2–78 years.

**Results:**

The findings indicate that: the majority of patients 57.4% were male, the most common age groups were 39–52 years old, and the mean age at diagnosis was 45 years. BCS is primarily caused by hepatic veins (HVs) thrombosis, which is observed in 18.03% of cases, and HVs are not seen in 55.73% of cases. Liver parenchymal enhancement appears heterogenous in 27.87%, while heterogeneously enlarged liver was seen in 24.59%, and cirrhotic in 14.75% of BCS patients. In comparison, 59.01 percent of BCS patients arrived without varices. Varices were observed in the splenorenal and gastroesophageal regions in 37.7% of cases. Ascites accounted for the majority of BCS complications 73.77%, with SM vein blockage and squeezed duodenum accounting for 3.27% of each complication. The likelihood of developing ascites increases with age, and it is most common in patients between the ages of 39 and 52 years. Patient age had the greatest effect on the development of ascites.

**Conclusion:**

The common features of BCS as revealed by contrast-enhanced CT of the liver are non-visible HVs, venous occlusion at either level of HVs or IVC, caudate lobe enlargement, heterogenous, normal or enlarged liver, collateral venous varices at the splenorenal and gastroesophageal region, and ascites.

## 1 Introduction

Budd-Chiari syndrome (BCS) consists of a heterogeneous group of conditions and manifestations defined as a severe vascular liver disease characterized by partial or total impairment of hepatic venous outflow [at the level of hepatic veins or hepatic portion of inferior vena cava (IVC)] without constrictive pericarditis or right heart failure. Most women with BCS are between the ages of 19 and 49, and the overall mean age ± stander deviation at the time of diagnosis is 40 ± 14 years (1–4). Alukal et al. (5) reported that over 19 years, the hospitalization rate of a patient with BCS increased from 4.96 per million in 1998 to 10.44 per million in 2017 in the United States of America (US). In the general population, BCS has a low incidence and prevalence, recent meta-analyses indicate that there are 1–1.4 new cases for every million residents annually (6–8). A high index of suspicion is required to make the diagnosis of BCS, which is still a condition that is mainly underdiagnosed. BCS should be taken into consideration when making a differential diagnosis in any case of unexplained portal hypertension as well as in all cases of acute and chronic liver disease (2). Budd Chiari syndrome can have several origins (causes), but the most prevalent one is the hypercoagulopathy state (thrombus seen in 87% of patients), which can be caused by congenital conditions like protein (C and S) deficiencies, myeloproliferative and polycythemia vera accounting for up to 50% of cases (9), paroxysmal nocturnal hemoglobinuria, pregnancy, and other illnesses (1, 10). A total of 25% are identified as having no underlying conditions, but BCS can arise secondary to tumor thrombus from renal cell carcinoma, liver tumors, especially HCC, and abscesses (1). Secondary BCS represents less than 1% of overall cases of BCS (11). Rarely, membranous web-like obstructions of the IVC that can spread to the hepatic veins might result in BCS; this disorder is more common in Asians and is most likely caused by genetic factors (12, 13). BCS is classified into two categories: primary and secondary, based on the cause and site of obstruction. While some researchers have divided BCS into fulminant, acute, subacute, and chronic categories, others typically classify them into acute, subacute, and chronic based on clinical manifestations (14, 15). The acute phase depends on the degree, level, duration, and distribution of obstruction, which can present clinical manifestations of acute liver failure; diagnosis based on liver enlargement (hepatomegaly) with or without ascites, which is not a common feature; and biopsy required in cases of diagnostic uncertainties (16). The chronic phase is characterized by the triad of hepatic and/or portal vein changes, fibrosis, and parenchymal remodeling (17). The number of HV involved, the thrombosis extension, and the impairment of the hepatic functional reserve because of increased intrahepatic sinusoidal pressure and congestion, which causes sinusoidal dilatation, liver congestion, and hepatomegaly, are all related to the presence and severity of the symptoms; as a result, a single venous blockage frequently presents with no symptoms, which is intended to increase portal hypertension, leading to extrahepatic and intrahepatic venous collateral formations (12, 18, 19). Various liver vasculatures can be affected by obstruction, either by primary or secondary causes: IVC, hepatic veins, and small veins, known as small vessels (BCS) (19). Small vessels of BCS develop at the level of the sinusoids and terminal hepatic venules (20), where the diagnosis using histological methods should be considered (19). Other investigators classify the BCS based on the level of obstruction (8, 10) and the duration of the disease (21). Most BCS are subacute and chronic. The clinical trial was present in over 60% of cases of abruptly developing abdominal pain, hepatomegaly, and ascites (3). These patients have a higher risk of developing hepatic necrosis, cirrhosis, and hepatic failure, which may require further liver transplantation (LT). LT is reported in about 10%–20% of BCS, mostly due to unsuccessful other methods of treatment or due to acute liver failure (12, 22). Other features include lower limb edema, GIT hemorrhage, fever, hepatic encephalopathy manifestations, and uncharacteristic jaundice (23). Kidney injury and hypercoagulable state in patients with nephrotic syndrome are associated with a poor prognosis (8, 24). Diagnosing BCS using radiological imaging is important to identify and confirm the obstruction of hepatic veins and/or IVC (25). In the past, hepatic cavography and venography were the gold standards in diagnosing BCS, until the introduction of color-doppler ultrasound (CDUS), cross-sectional imaging as CT, and magnetic resonance imaging (MRI) made the non-invasive procedure and diagnosis possible. For clinically suspected patients with BCS, the guideline recommended CDUS as the first line of examination followed by cross-sectional imaging CT and MRI to confirm the diagnosis (12, 19, 26). Also, CT and MRI were used to assess the thrombus extensions, characterize hepatic nodules, the degree of disease, plan treatment interventions, diagnosis of tumors, and liver parenchymal evaluations (2, 12, 27). Imaging characterization and diagnosis of BCS depend on the stage of the disease (28). Imaging characteristics include venous collaterals, stagnant or inverted venous flow, blockage or compression of the hepatic veins and/or the IVC (called direct signs); morphological features secondary to direct features include hypertrophy of unaffected segments (e.g., caudate lobe) and atrophy of affected segments leading to portal hypertension and other features such as heterogeneity of liver parenchyma, splenomegaly, and ascites in addition to delaying the nodule formation (13, 28). MRI and ultrasound with vascular analysis are essential to confirm a positive diagnosis; angiography is often performed as the first step before endovascular treatment (13). US with a sensitivity of 85% exhibits splenomegaly, ascites, an enlarged caudate lobe, and heterogenous liver echogenicity (29). Contrast-enhanced computed tomography (CECT) provides evaluation and characterization of ascites, hepatic vein and IVC patency, and caudate lobe hypertrophy, in addition to vascular collaterals, edema, and various abdominal complications related to this disease in acute and chronic conditions (30). The full length of the IVC can be seen by MRI, which also helps to distinguish between acute, subacute, and chronic BCS (31). The two components of BCS management are decompression of the hepatic venous outflow and lifelong anticoagulation (due to the underlying procoagulant condition) (endovascular or surgical) (19). Angioplasty with a success rate of more than 90% as reported by different authors to resolve occlusion and stenosis in both IVC and hepatic web (32) also Transjugular intrahepatic portosystemic shunt (TIPS), which indicated when liver damage (congestive) resulted from decreased (or complete obstruction) venous and arterial blood supply leads to collateral (slow) development. As shown with surgical portosystemic shunts, TIPS leads to improved liver function and blood flow (32, 33). The outcome of BCS as reported by Sharma et al. (19), Zhang et al. (34) stated that; the outcome dramatically improved in recent decades, with 1, and 5 years survival rates of 92% and 76.4%, respectively for endovascular treatment, 95.9% and 88.6%, respectively for angioplasty, 87.3% and 72.1% for TIPSS. Since BCS is thought to be less common in Sudan, this study sought to determine what characteristics all BCS patients had in common. This is because BCS can be mistakenly identified as chronic liver disease under various circumstances. Due to Sudan’s limited access to radiological resources, particularly MRI and CT, an additional exclusion diagnosis of this disease has been made depending on other methods of diagnosis. CT is used to characterize a wide range of abdominal diseases, and it is through this that Budd Chiari is incidentally detected in patients with known diseases. This study aimed to describe the common radiological features of BCS by triphasic CT abdomen in the Sudanese population, as well as the identification of common associated hepatobiliary findings, vascular abnormalities, and complications.

## 2 Materials and methods

### 2.1 Study design and participants

The 61 patients in the retrospective, cross-sectional descriptive study and a few presented case reports from Sudan, whose ages vary from 2 to 78 years old and whose mean age is 43.5 years old, completed successful Triphasic CT examinations.

### 2.2 Inclusion criteria

The patient’s condition, including whether or not the patient has a known case of Budd Chiari syndrome or whether the patient’s CT scan revealed an incidental finding, determined the patient selection criteria for the 16-slice Optima 520 General Electric (GE) machine used at various Sudanese hospitals and diagnostic centers, particularly a Kuwaiti specialized hospital, during the period from March 2022 to March 2023.

### 2.3 Exclusion criteria

A total of 2,880 contrast-enhanced abdominal CT scans were performed during the study period; 2,819 of these cases were excluded based on the final CT result, which was either normal or indicated that the patient had other diagnoses besides BCS.

### 2.4 Scanning protocol and image interpretation

The suspicious patient was presented to the radiology department with symptoms of right upper quadrant pain, ascites, abdominal pain, and liver cirrhosis. The patient underwent a triphasic CT abdomen procedure; it is recommended that the patient fast for at least three hours for solid food and that negative oral contrast (oral water) be taken 30–45 min before beginning any CT procedures. An axial non-contrast CT scan with a 5 mm slice thickness at 5 mm intervals is performed from the lower chest up to the iliac crests to visualize abnormalities in the liver and upper abdomen. A total of 1–1.5 L of water is considered a negative (pure water only) contrast to delineate the upper abdomen, including the stomach and upper GI system for normal anatomical and pathological differentiation or any feature of the calcifications or atherosclerotic changes that may affect the IVC or the great arteries as well as the muscular arteries. The arterial phase was obtained 20–25 s after bolus tracking with a contrasting dose of 1.5 ml/kg–5 ml/sec injection rate using an automatic injector to visualize the arteries in the upper abdomen to the bifurcation of the aorta. A portal-venous phase was performed 60–75 s after the start of the contrast injection from the lower chest to the ischial tuberosity, and delayed phases were taken for the liver 6–10 min after the contrast injection. CT and Doppler US are the diagnostic tools for diagnosing BCS in Sudan, once the clinical indication and scanning process have been identified, the consultant radiologists interpret the patient’s images to determine the final diagnosis. There are no agreed-upon single diagnostic criteria for describing BCS radiological features in Sudan. Patient clinical indications, inability to identify hepatic veins in CT, and no Doppler signal in color Doppler or loss of phasicity were commonly unanimous radiological features to describe BCS. According to the literature, the differential diagnosis of BCS includes right-sided heart failure, metastatic liver disease, alcoholic liver disease, granulomatous liver disease, Alpha-1 antitrypsin deficiency, infectious hepatitis, congestive heart failure, hemochromatosis, biliary atresia, congenital hepatic fibrosis, Niemann-Pick disease type C, Cystic fibrosis, inborn errors of metabolism, hemochromatosis and drug-induced hepatitis (35–39).

### 2.5 Data collection

Age, gender, indication, liver disease, liver features of the BCS, venous circulation status, and other features including splenic appearance and complications, presence of venous varices, and related complications are among the variables included in the data collection. All information was gathered using the final pictures and the consultant radiologist’s final report.

### 2.6 Statistical analysis

Data was displayed using frequency tables. All statistical analyses were conducted using SPSS (version 27.0, IBM, New York, United States) software.

## 3 Results

### 3.1 Results summary

This study included 61 patients diagnosed with BCS using CT examination. Gender distribution is more prevalent for males (57.4%) than females (42.6%). The average age at the time of diagnosis was 45 years, with the most affected age group being 39–52 years old (23%), followed by 27–39 years old (20%). The main indications were abdominal pain (57.4%), and jaundice (36.06%) which is more commonly linked to vomiting and less commonly associated with general edema, which was observed in 4.91% of cases, Abdominal distention was noted in just 1.6% of cases. Direct hepatic manifestations included an enlarged caudate lobe (47.54%), heterogeneous (nutmeg) liver enhancement (27.86%), and enlarged liver (24.59%). Ascites were common (73.77%) of cases, representing cardinal association with BCS. The overall CT findings for BCS patients were hepatic vein thrombosis (18.03%), followed by intrahepatic venous collaterals (18.03%), begin-looking liver nodules (14.75%), HCC (9.84%), PV and SMV thrombosis (6.56%), and chronic liver disease along with a thick wall Gallbladder with stone (6.56%). The venous pattern showed no visible hepatic veins in 55.73% of cases, narrow (barely seen) HVs (18.03%), compressed IVC (6.56%), PV dilatation (8.19%), while dilated splenic and SM veins seen in (3.27%). Notably, 59% of patients had no venous varices, commonly seen in splenorenal and gastroesophageal regions (37.70%). One of the most common CT findings as a complication of BCS was ascites as seen in 73.77% of cases, while no complications were reported in (18.03%) of the patients.

## 4 Discussion

**FIGURE 1:**
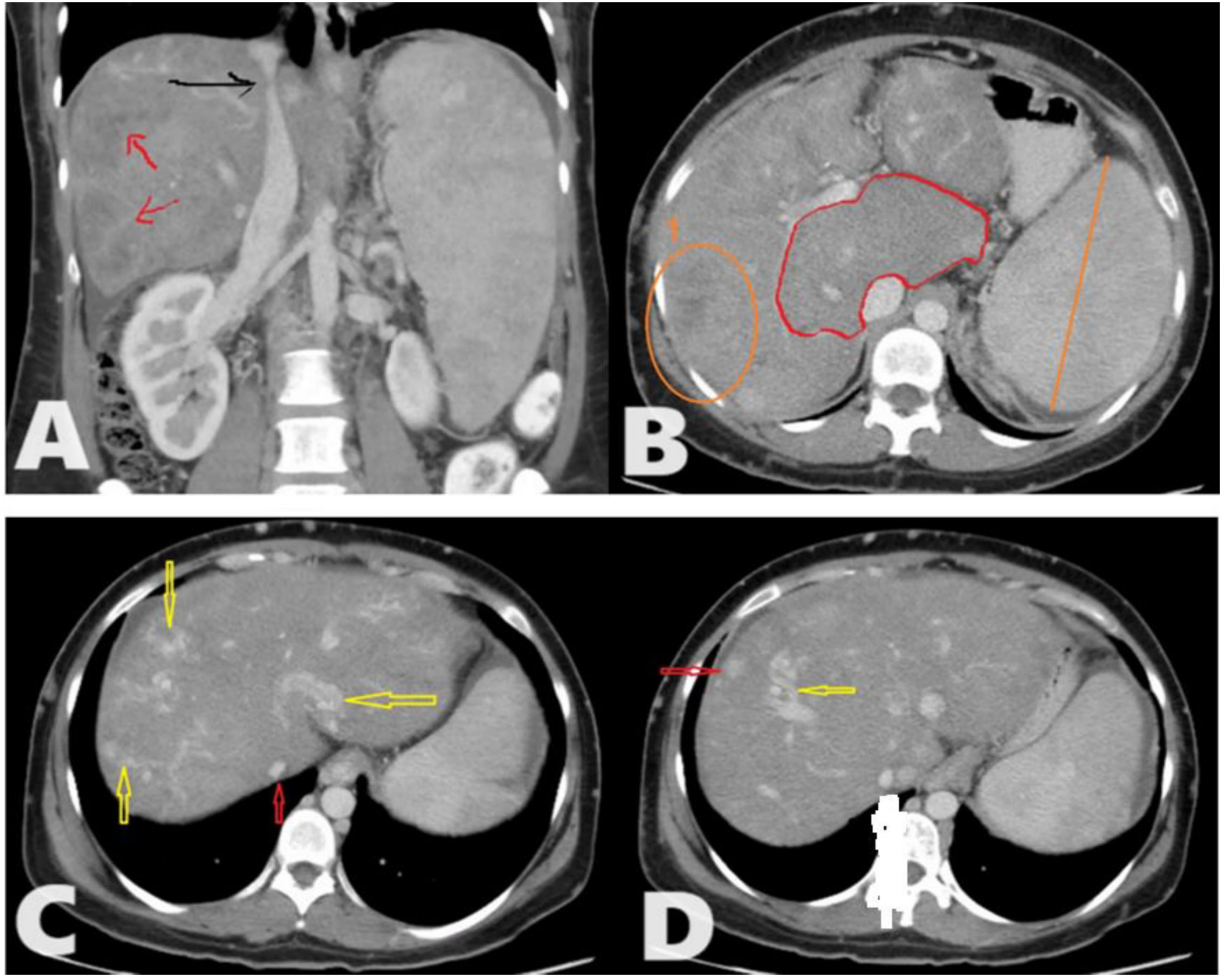
A total of 35 years female presented to the radiology department with chronic abdominal pain. Triphasic computed tomography (CT) abdomen reveals; **(A)** Chronic Budd-Chiari syndrome (BCS) manifested as Enlarged liver with heterogeneous enhancement and a patchy hepatogram with areas of hypoperfusion (Red arrows). The hepatic part of inferior vena cava (IVC) shows a focal stenosis which may indicate the presence of an IVC web (Black arrow). **(B)** The caudate lobe is disproportionately enlarged compared to the rest of the liver. No IVC/portal vein thrombosis. **(C)** There are multiple intrahepatic collateral vessels, some of which drain directly into the IVC (yellow arrows) and narrow IVC (red arrow) with no enhancement of the hepatic veins (HVs) (inability to identify HVs). **(D)** There are multiple enhancing nodules spread throughout the liver (Red arrow) as an example.

**FIGURE 2:**
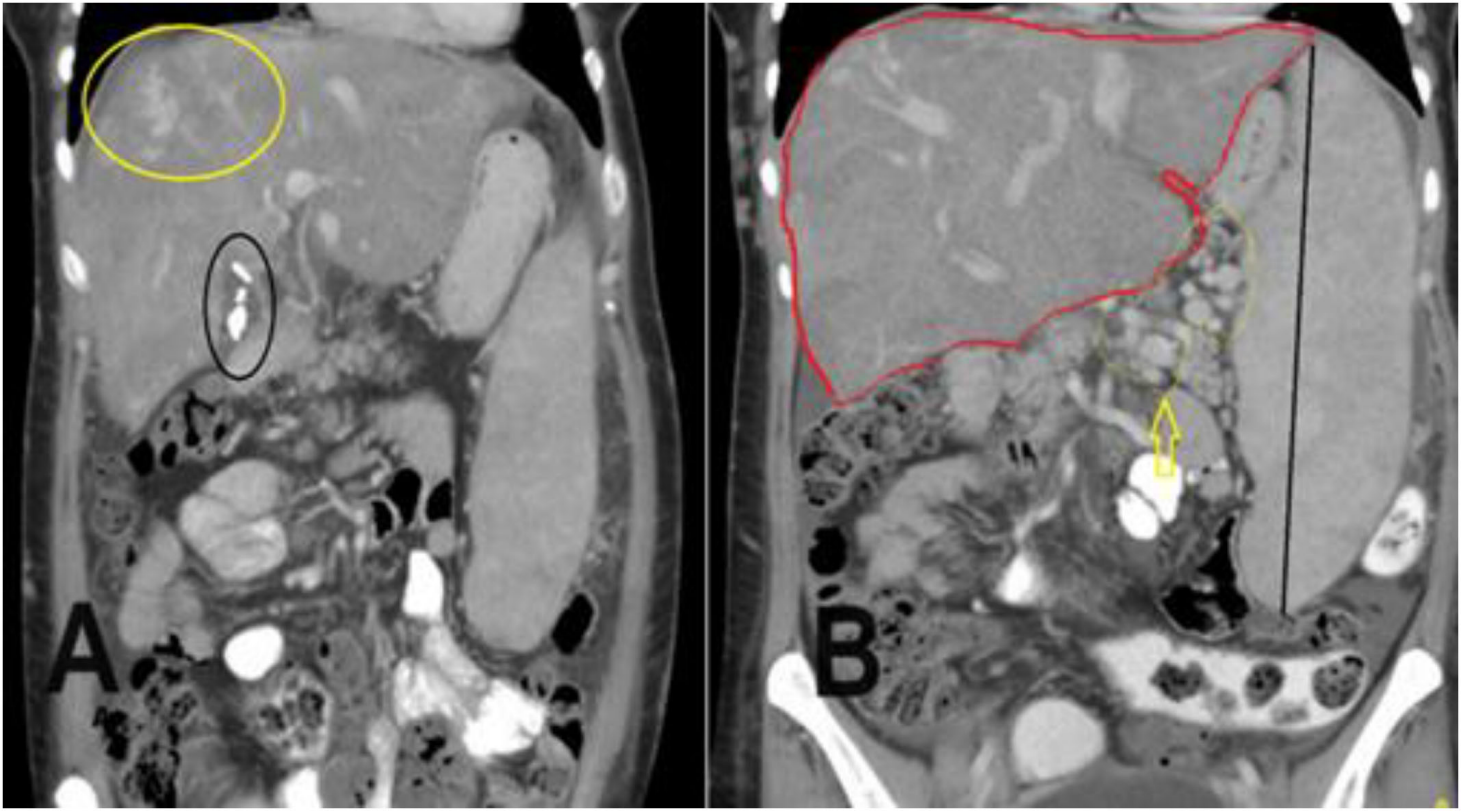
A coronal section of the portal venous phase of the computed tomography (CT) abdomen for the previously mentioned patient as in Figure 1 demonstrates **(A)** incidental finding of cholecystectomy clips and fluid in the gallbladder fossa indicating recent cholecystectomy. **(B)** Hepatomegaly and a huge spleen are consequences of Budd-Chiari syndrome (BCS), splenic varices (yellow arrow), and multiple collateral veins in the abdominal wall.

Digital Subtraction Angiography (DSA) is considered a “gold standard” modality to diagnose BCS, but its invasive procedure involving the use of contrast materials and ionizing radiation (42), Doppler US is considered the first modality of choice for initial investigation when BCS is suspected (43), but it highly depends on the operator’s experiences; ultrasound is also affected by excessive ascites and bowel gases (42, 44). CT is widely used in BCS diagnosis, it’s used to evaluate liver parenchyma, HVs, IVC, and venous collaterals (45). However, CT is considered less accurate in the detection of venous abnormalities compared with CDUS and MRI (12). Also, the use of contrast agents may lead to allergic reactions, and possibilities of contrast-induced nephrotoxicity were the major drawbacks (46). MRA is an alternative technology used for vascular assessment in BCS without the use of contrast materials (47). It is also used for the classification of BCS patients (48). A high rate of heartbeats, massive ascites, and uneven breathing were factors of misdiagnosis in MRA of BCS patients (42). The sensitivities and specificities for Doppler in the diagnosis of BCS were 89% and 68%, 89% and 72% for CT, and 93% and 55% for MRI, respectively, indicating higher MRI sensitivity and higher CT specificity compared to both modalities. MRI yielded an AUC of 90.8% compared with 88.4% for CT and 86.6% for Doppler ultrasound (49). This research was conducted to characterize and identify the common feature of Budd Chiari syndrome in the Sudanese population using CT scan, CT and Doppler US are the available and most used diagnostic modalities in Sudan to identify the BCS features, contrast-enhanced liver CT is readily used for abdominal and liver disease scans more frequently than MRI, due to the limited number of MRIs in Sudan. Figures 1, 2 give a clear picture of BCS features and their related complications. Different age groups were investigated; in which BCS affects both children and adults, a median age of 10.5 (2–16 years) was identified (50) which is commonly associated with abdominal distention in more than 86% of children and upper GI bleeding in more than 36% of patients as common primary indication and findings as detected by Ultrasound and MRI in few patients. BCS is rare in children and accounts for more than 16 percent of pediatric liver illnesses in Asia, but less than 0.1 percent in Western nations (51), our present research indicates that the most common age group for affected children was 02–14 years, same results also indicated by different authors including (50, 52). This study focused on different ages as adults were more frequently included resulting from a random collection of data, a common age group affected in adult patients at the time of diagnosis was 39–52 years, as also seen by Ollivier-Hourmand et al. (53) in France, who stated the mean age was 40 ± 14 years. A total of 45 years mean age as in (54). BCS affects both males and females, but in this study, the most affected gender was male at 57.4%, while females represented 42.6% of the total sample distribution. This result is quite different from a variety of studies reported by different authors, as females were commonly affected rather than male gender, as in (55, 56), Female predominance is reported in the West (67%) (57). But also, slight male predominance was reported in India (58) and Japan (59). Dilawari et al. (58) stated that males are commonly associated with IVC obstruction, while females are commonly associated with Hepatic Viens (HV) obstructions. Manifestation of such clinical signs depends on the degree of hepatic vein involvement and portal vein, as in Figure 1A. This study showed that the most common signs and manifestations of BCS were abdominal pain seen in 57.4% of patients, followed by jaundice associated with edema and/or vomiting in 42% of patients. Less frequent signs were seen as abdominal distention as a result of intra-abdominal bleeding in 1.63% of the study populations. Different authors report that hepatomegaly, ascites, abdominal discomfort, and the existence of dilated superficial abdominal wall veins are the main characteristics of BCS (13, 60). To distinguish between vascular and parenchymal liver disease was commonly diagnosed using contrast-enhanced CT abdomen with varying contrast timing. Computed tomography has recently been shown to be an excellent modality of choice for the characterization, classification, and staging of BCS, especially for chronic stages of disease (13). The liver parenchymal appearance showed various patterns of enhancement, with a heterogenous (nutmeg) liver seen in most of the patients 27.87%. In comparison, an enlarged liver also with heterogeneous contrast enhancement was identified in more than 24% of the CT scan results. Liver cirrhosis is a remarkable characteristic of different chronic liver diseases, especially hematogenous-associated diseases. BCS is a rare cause of liver cirrhosis that can be distinguished from other causes of cirrhotic disease by normal values of liver function and prominent portal hypertension (PH) (61). Cirrhosis caused by BCS seen shows 14.8% of patients with cirrhotic heterogeneous liver, as in Table 1. As we previously mentioned, causes of BCS are most commonly due to vascular causes, HV thrombosis due to hemodynamic disorders, or secondary due to tumor thrombus from RCC or HCC (1, 9, 10). Hepatic vein thrombosis was seen in 11 patients (18.03%), A CT scan of the patient with BCS reveals a wide range of findings including biliary diseases, hepatic lesions either benign or malignant lesions, chronic liver diseases such as viral or non-viral conditions, gallbladder stone associated with wall thickening seen in 6.56% of BCS patients, also the background of the shrunken liver with an irregular outline in 4.92%, liver cyst or gallbladder cancer, IVC thrombosis associated with hemangioma, chronic hematological disease, hepatic vein obstruction with tumoral thrombus in IVC, chronic liver disease associated with multiple nodules, and chronic hepatic vein thrombosis as presented in Table 2. Collectively these (CT findings) related to BCS were seen in more than 52% of study populations compared to more than 87% of patients, as reported by Denninger et al. (9); see Table 2. In 80%–91% of instances, the caudate lobe enlarges as a result of distinct venous outflow (62, 63). A “flip-flop” pattern develops during the portal venous phase, with modest attenuation of the liver’s core region due to washout. A different form of venous changes and appearance in computed tomography were identified in this study. HVs were not identified in more than half of the patients diagnosed with BCS (55.73%), narrowing or barely seen hepatic veins, slit-like (compressed) IVC, portal dilatation, dilated celiac, and SM vein; all these changes were seen in Sudanese populations, as presented in Table 3. BCS is linked to varying degrees of variceal alterations because of venous involvement. Commonly, no variceal changes are seen with BCS in a significant percentage of the patients including more than 59% of them, but due to these obstructive changes and increased vascular permeability. The most common site of varices is the lienorenal and gastro-esophageal region seen in 37.7% of patients with BCS and varying degrees of involvement in gastric and esophageal veins. Still, many cases were not associated with varices in more than 59% of patients, see Table 4. Naganuma et al. (64) reported that; BCS is one of the main causes of intrahepatic and abdominal collaterals. Hepatomegaly and ascites are caused by acute blockage of the hepatic venous outflow (attenuation/thrombosis of HVs and/or IVC). Venous thrombosis causes the portal venous blood influx to be delayed or reversed as well as an increase in sinusoidal pressure. In the acute form, hepatic necrosis happens quickly, and venous collaterals have not yet formed (13, 63). This study reported that more than 73.7% of patients presented with ascites (associated with an umbilical hernia in 1.63%) of the patients. In addition to other rare complications such as compressed duodenum, splenic, and SM vein occlusion, as shown in Table 1. Ascites is commonly associated with older ages, especially 39–52 years as the peak age of patients is strongly linked to such complications. Radiologic investigations of BCS are non-invasive procedures. US allows the assessment of hepatic venous flow and heterogeneity, while CT and MRI can depict HVs thrombosis and IVC occlusion or compression.

**TABLE 1:** Determined the liver’s direct appearance and other indirect abdominal results from the computed tomography (CT) abdomen.

Categories	Frequencies	%
Liver appearance (direct liver CT findings)*		
caudate lobe enlargement	29	47.54
heterogeneous (nutmeg) liver enhancement	17	27.87
hepatomegaly with heterogeneous liver enhancement	15	24.59
Indirect abdominal finding*		
Ascites	45	73.77
Ascites and umbilical hernia	1	1.63
Splenic and SM vein occlusion	2	3.27
Compressed duodenum	2	3.27
No complications	11	18.03
Total	61	100.0

*n = 61 for both direct and indirect findings.

**TABLE 2:** Hepatobiliary computed tomography (CT) finding in a patient with Budd-Chiari syndrome (BCS).

Hepatobiliary findings	Frequency	Percent
Hepatic vein thrombosis	11	18.03
Intrahepatic venous collaterals	11	18.03
Benign-looking liver nodules	9	14.75
Biliary disease	2	3.27
Chronic liver disease, gallbladder stone, and GB wall thickening	4	6.56
Background of shrunken liver and irregular outline	3	4.92
Liver cyst and gallbladder cancer	2	3.27
IVC thrombosis and hemangioma	2	3.27
Hepatic vein obstruction and tumoral thrombus IVC	2	3.27
Multiple nodules, and Chronic liver disease	2	3.27
Liver HCC	6	9.84
Ca head of pancreas	2	3.27
Choledochal cyst	1	1.63
PV and SMV thrombosis	4	6.56
Total	61	100

**TABLE 3:** Frequency distribution of venous patterns of appearance related to Budd-Chiari syndrome (BCS) in computed tomography (CT) scan.

Venous abnormalities in BCS	Frequency	Percent
Narrowing of hepatic veins	11	18.03
Slit-like (compressed) IVC	4	6.56
Portal vein dilatation	5	8.19
Not seen hepatic veins	34	55.73
Dilated splenic and SM veins	2	3.27
Compressed IVC with barely seen HVs	5	8.19
Total	61	100

**TABLE 4:** Associated variceal changes as seen in the computed tomography (CT) abdomen.

Varices	Frequency	Percent
Lienorenal (splenorenal) and gastroesophageal varices	23	37.7
No varices	36	59.01
Gastric and esophageal	1	1.63
Gastric	1	1.63
Total	61	100.0

### 4.1 Limitations

Since most patients were seen as outpatients and came for radiological CT and/or Doppler ultrasound procedures based on information provided by the referring physician, it is challenging to follow up with the patients. Also, there is no further investigation of the patterns of BCS.

The diagnosis of BCS was not based on a golden standard such as DSA.

This research described the radiological features based on CT studies; there was no clear differentiation made between patients with acute and chronic BCS in the study, and the severity of the condition was not categorized.

## 5 Conclusion

Although few case reports have been conducted, as presented in Table 5 there aren’t many publications in this field. This is the first study of its kind that presents the features of BCS as presented to the radiology department in Sudan. Identification of thrombosis and/or stenosis of one or more HVs or the upper section of the IVC on imaging is necessary for the diagnosis of BCS. The most common type of thrombosis is persistent, involves many HVs, and manifests as non-visible venous segments or fibrous tracts. CT is one of the most powerful tools that can be used in primary detection, staging, and characterization of BCS, as well as thrombosed or narrow veins in addition to characterization of early and late complications of BCS as well as liver parenchymal appearance. According to this study, BCS in the Sudanese population is seen more in males, commonly presenting with abdominal pain; the most encountered CT findings were barely visible or inability to identify hepatic veins, enlarged heterogeneously enhancing liver, and ascites, with less frequently observed variceal changes.

**TABLE 5:** Status of Budd-Chiari syndrome (BCS) in Sudan as presented in the literature.

References	Description	Patient conditions	Methods of radiological diagnosis
Obed et al. (40)	Presented a case report of a 47 years female with acute on chronic liver failure with subacute BCS triggered by bacterial pneumonia, underwent successful liver transplantation	Rare co-existence of pathologies, finally diagnosed with antiphospholipid antibody syndrome, presented with liver failure, acute kidney failure, acute respiratory failure, and encephalopathy stage IV	In addition to liver transplant investigations, CT Angio for CAP to assess the BCS severity also status of IVC was conducted which resulted in severe narrowing of IVC with occlusion of main HVs along with ascites and bilateral pleural effusion
Sawaqed et al. (41)	Presented case report of BCS following COVID-19 infection	Clotting disorder associated with COVID-19 leads to BCS, presents severe epigastric and RUQ pain, hepatomegaly, and progressive abdominal distention	Different hematological, immunological, and radiological tests. US reveals hepatosplenomegaly with free pelvic fluid. Triphasic CT reveals hepatosplenomegaly and HVs Occlusion. Doppler ultrasound also confirmed the diagnosis

## Data Availability

The raw data supporting the conclusions of this article will be made available by the authors, without undue reservation.
